# Facilitators and barriers to *in vitro* diagnostics implementation in resource-limited settings: A scoping review

**DOI:** 10.4102/phcfm.v15i1.3777

**Published:** 2023-02-03

**Authors:** Monica Ansu-Mensah, Desmond Kuupiel, Emmanuel A. Asiamah, Themba G. Ginindza

**Affiliations:** 1 Department of Public Health Medicine, Faculty of Health Sciences, University of KwaZulu-Natal, Durban, South Africa; 2 The University Clinic, Sunyani Technical University, Sunyani, Ghana; 3 Faculty of Health Sciences, Durban University of Technology, Durban, South Africa; 4 Centre for Infectious Diseases Epidemiology Research Unit (CIDERU), College of Health Sciences, University of KwaZulu-Natal, Durban, South Africa; 5 Department of Medical Laboratory Sciences, School of Allied Health Sciences, University of Health and Allied Sciences, Ho, Ghana

**Keywords:** facilitators, barriers, essential *in vitro* diagnostics, primary healthcare facilities, LMICs

## Abstract

**Background:**

The World Health Organization (WHO) developed the model list of essential *in vitro* diagnostics (EDL) to guide countries to develop and update point-of-care (POC) per their disease priorities. The EDL includes POC diagnostic tests for use in health facilities without laboratories; however, their implementation might face several challenges in low- and middle-income countries (LMICs).

**Aim:**

To identify facilitators and barriers to POC testing service implementations in the primary health care facilities in the LMICs.

**Setting:**

Low- and middle-income countries.

**Methods:**

This scoping review was guided by Arksey and O’Malley’s methodological framework. A comprehensive keyword search for literature was conducted in Google Scholar, EBSCOhost, PubMed, Web of Science and ScienceDirect using the Boolean terms (‘AND’ and ‘OR’), as well as Medical Subject Headings. The study considered published articles in the English language from 2016 to 2021 and was limited to qualitative, quantitative and mixed-method studies. Two reviewers independently screened the articles at the abstract and full-text screening phases guided by the eligibility criteria. Data were analysed qualitatively and quantitatively.

**Results:**

Of the 57 studies identified through literature searches, 16 met this study’s eligibility criteria. Of the 16 studies, 7 reported on both facilitators and barriers; and the remainder reported on only barriers to POC test implementation such as inadequate funding, insufficient human resource, stigmatisation, et cetera.

**Conclusion:**

The study demonstrated a wide research gap in facilitators and barriers, especially in the general POC diagnostic test for use in health facilities without laboratories in the LMICs. Extensive research in POC testing service is recommended to improve service delivery.

**Contribution:**

This study’s findings contribute to a few works of literature on existing evidence of POC testing.

## Introduction

The battle against communicable and noncommunicable diseases (NCDs) has recently become the highest priority in the low- and middle-income countries (LMICs), especially in the World Health Organization (WHO) Africa Region.^1,2,3,4,5^ The majority of the top 10 causes of death occurring in sub-Saharan Africa (SSA) are from communicable diseases.^3^ Mention could be made of malaria, human immunodeficiency virus (HIV) and tuberculosis (TB) death.^4^ The WHO global malaria report for the year 2020 showed 241 million cases and 62 700 deaths, out of which 95% of the cases and 96% of deaths occurred in the WHO Africa Region. Again, about 10m cases and 1.5m deaths are recorded every year for TB with the larger proportion occurring in the LMICs.^6^ Sub-Saharan Africa has over the past decade experienced a surge in diabetes and hypertension among these countries with 80% premature deaths from NCDs.^1^ In an attempt to combat the disease burdens, the Sustainable Development Goals (SDGs) seek to ensure healthy lives and promote well-being at all ages by 2030.^7^ This partly necessitated the introduction of the WHO model list of essential *in vitro* diagnostics (EDL), as the basis for strengthening diagnostic testing capacity and increasing access to *in vitro* diagnostics (IVDs). The EDL offers guidance to countries on methods to develop, update and prioritise the IVDs.^8^ The WHO’s EDL provides a range of tests for general and disease-specific IVDs mostly in point-of-care (POC) form for use in healthcare facilities with or without laboratories.^8^ Tier 1 facilities refer to primary care settings with healthcare professionals but no trained laboratory personnel, self-testing or low resource settings.^8,9,10,11,12^ Point-of-care diagnostics refer to advanced technological-based medical devices for testing, screening and monitoring diseases in services near patients or clients.^13,14^ Point-of-care diagnostics have shown to be a useful tool for improving disease diagnosis and treatment globally.^15,16^ Evidence showed POC testing has improved antenatal HIV screening in sub-Sahara Africa.^3^ In resource-limited settings, POC technologies have become reliable and very important by providing healthcare providers with the easiest, most convenient and most accurate way of decision-making on diagnosis and treatment.^3,14,17,18,19,20,21,22,23^ The quality of POC for limited-resource settings according to the WHO should be designed to meet the following benchmarks: affordable, sensitive, specific, user-friendly, rapid or robust, equipment-free, and delivered to those who need it (ASSURED).^8,24,25,26,27^

Despite the benefits derived from POC testing, there are challenges with its implementation, which hinder accessibility for many patients in the LMICs.^28,29^ Implementation and sustainability of POC testing in resource-limited settings are feasible when potential barriers are addressed.^30^ Barriers to POC testing implementation may include challenges making POC testing service implementation difficult. Examples of these include low availability, low stock levels, procurement issues, poor supply chain management, funding, human resource capacity and many others.^21,24,27,31,32,33^ Facilitators of POC testing are motivators or factors which contribute to POC testing implementation. For instance, effective regulations on quality and training enable the successful implementation of POC testing in healthcare facilities.^34^

A wide research gap in the general POC test for use in a health facility without laboratories suggests presumptive treatment and poor health outcomes in many LMICs. Therefore, the need to investigate the barriers and facilitators of POC test implementation of general POC diagnostic testing services is of utmost importance.

## Methods

We adopted Arksey and O’Malley’s framework as a guide to conduct this scoping review. The preferred reporting items for systematic reviews and meta-analyses extension for scoping reviews checklist were used to report this study.^35^

### Identifying the research question

The research question for this study was: To date, what evidence exists on facilitators and barriers to implementation of the WHO EDL for use in tier one healthcare facilities in LMICs? Table 1 shows the framework (population, content and context [PCC]) used to determine the suitability of the review question.

**TABLE 1:** Population, content and context framework for defining the eligibility of the studies for the primary research question.

Population	POC tests/WHO EDL for tier 1 facilities: This will include general IVDs for community and health settings without laboratories such as (Albumin, Bilirubin, Glucose, etc.^9^ and disease-specific IVDs for use in health settings without laboratories e.g. (Malaria, syphilis, HIV infection, etc.^9^
Concept	Facilitators to implementation: facilitators are things, which give room to POC test implementation e.g. Adequate funds, and adequate human resources.^36^ Barriers to implementation: barriers refer to challenges to implementing the POC test (unavailability, no definition of policies, lack of funding opportunities, inadequate human resource capacity, poor supply-chain).^36^
Context	Low- and- middle-income countries: This will include countries classified as low-income, lower-middle-income, and upper middle-income by the World Bank.^37^

*Source*: Tricco AC, Lillie E, Sarin W, et al. PRISMA extension for scoping reviews (PRISMA-ScR): Checklist and explanation. Ann Intern Med. 2018; 169(7):467–473

POC, point-of-care; IVDs, *in vitro* diagnostics; HIV, human immunodeficiency virus; WHO EDL, World Health Organization essential *in vitro* diagnostics.

### Literature search

With a date limitation of 2016–2021, we searched five electronic databases (Google Scholar, Academic Search Complete via EBSCOhost, PubMed, Web of Science and ScienceDirect) for relevant studies (Appendix 1). We used a combination of the following keywords: ‘facilitator’, OR ‘enablers’ AND ‘barriers’ AND ‘point-of-care testing services’, AND ‘point-of-care diagnostics services’, AND ‘*in vitro* diagnostics’, AND ‘implementation’, AND ‘lower-and-middle income countries’ OR ‘LMICs’. Medical subject headings were applied in the search strategy. Limitations on language and study design were removed.

### Eligibility criteria

Articles published only in the English language were included subject to the eligibility criteria. Such articles also had to be written in at least one of the LMICs on facilitators and barriers and focus on either POC testing services or *in vitro* diagnostics in primary health care (PHC) facilities. This review was limited to primary study designs (qualitative, quantitative and mixed-methods study). We excluded all articles published before the year 2016.

### Study selection

The databases’ search and the title screening were conducted using the eligibility criteria. A clean library was shared with the review team after all duplicates were removed. Two authors independently screened abstracts and full articles using tools pilot-tested by the review team. The review team discussed all discrepancies that arose at the abstract screening stage between these two authors until a consensus was reached. Then, the last two authors addressed the discrepancies during the full-text screening phase.

### Charting the data

We extracted the following: author and publication year, the country where the study was conducted, study design, study setting, study population, type of POC test, type of barrier of POC diagnostics testing, facilitators to POC testing implementation, type of general IVDs test, and type of disease-specific diagnostic test. We also extracted the findings relevant to answering the review question using a deductive approach. To ensure the credibility and accuracy of the study finds, Monica Ansu-Mensah and Desmond Kuupiel independently abstracted the data with TGG: Themba G. Ginindza acting as the arbitrator.

### Collating and summarising the results

Thematic analysis was conducted following the data extraction. The data were collated into themes and a summary of the study outcomes was reported in a narrative form.

### Ethical considerations

This article followed all ethical standards for research without direct contact with human or animal subjects.

## Results

**FIGURE 1:**
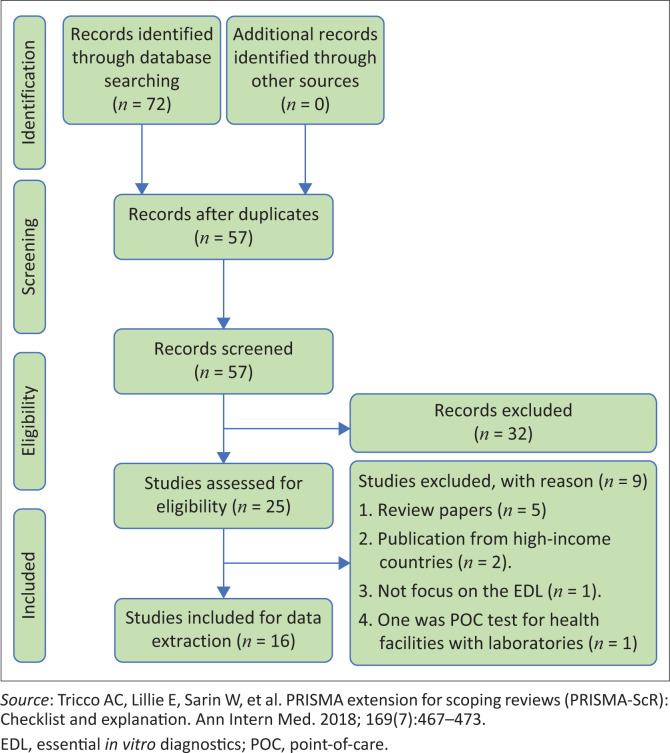
PRISMA flow diagram.

Of the 72 eligible articles obtained from the databases search, 16 duplicates were removed. Out of the remaining 57 articles screened, 32 were excluded at the abstract screening stage. A further 25 articles were removed during the full-text screening phase. Finally, 16 articles remained for data extraction and analysis. The reasons for exclusion following the full-text screening were the following: five were review papers^24,38,39,40,41^; two were conducted in high-income countries^42,43^; one article focused on a POC test not included in the WHO EDL^44^; and one reported on a POC test for health facilities with laboratories^45^ (Figure 1).

### Characteristics of the included studies

Out of the 16 included articles for this study, five (31%) reported from South Africa,^46,47,48,49,50^ three (19%)^4,27,51,52,53,54^ reported from Kenya and Ghana respectively, one (6%) each was conducted in Papua New Guinea (PNG),^55^ Burkina Faso,^4^ Malawi,^56^ there were two multi-country studies (Zambia and Malawi),^57^ and (Brazil, Bulgaria, China, Macedonia, Malaysia, Peru, Serbia, South Africa, Turkey, Burma, Egypt, Georgia, India, Indonesia, Kenya, Nigeria, Pakistan, PNG, Vietnam, Cambodia, Mali, Uganda and Zimbabwe).^58^ Of the 16 included articles, 63% (*n* = 10) were qualitative studies^4,47,48,51,52,53,56,57,58,59^; 25% (*n* = 4) were cross-sectional surveys^27,46,48,54^; and approximately 6% (*n* = 1) each was a mixed-method^55^ and an experimental study^49^ (Table 2).

**TABLE 2:** Study characteristics.

Author & year	Country of Study	Type of POC test	General or Disease-specific	Study design
Chamane et al, 2020^46^	SA	HIV	Disease-specific	Cross-sectional
Hecke et al, 2019^48^	SA	POC tests (unspecified)	Unspecified	Qualitative
Kuupiel et al, 2019^54^	Ghana	HBV, HCV, glucose, blood typing, urinalysis test, HB, syphilis	Both general and disease-specific	Cross-sectional
Maeharia et al, 2020^53^	Kenya	HIV	Disease-specific	Qualitative
Reddy et al, 2020^47^	SA	HIV	Disease-specific	Qualitative
Rao et al, 2020^5^	SA	HIV	Disease-specific	Cross- sectional
Hershow et al, 2019^57^	Malawi and Zimbabwe	HIV	Disease-specific	Qualitative
Mohamed et al, 2020^55^	PNG	HIV	Disease-specific	Mixed-method
Wexler et al, 2021^52^	Kenya	HIV	Disease-specific	Qualitative
Ginderdeuren et al, 2019^49^	SA	TST	Disease-specific	Experimental study
Palmer et al, 2020^4^	Ghana	Malaria	Disease-specific	Qualitative
Wexler et al, 2019^51^	Kenya	HIV	Disease-specific	Qualitative
Bocoum et al, 2017^59^	Burkina Faso	Syphilis	Disease-specific	Qualitative
Reipold et al, 2017^58^	23 Multi countries (LMICs)	HBV, and HCV	Disease-specific	Qualitative
Maddox et al, 2017^56^	Malawi	HIV/syphilis	Disease-specific	Qualitative
Kuupiel et al, 2019^27^	Ghana	HB, blood glucose, HIV, syphilis, HBV, malaria, urine pregnancy, urine protein	Both general, and disease-specific	Cross-sectional

HIV, human immunodeficiency virus; LMICs, low- and middle-income countries; PNG, Papua New Guinea; POC, point-of-care; SA, South Africa; HBV, hepatitis B virus; HCV, hepatitis C virus; HB, haemoglobin; TST, tuberculin skin test.

## Study findings

Of the 16 included studies, 7 studies reported on both facilitators and barriers to POC testing implementation.^47,51,52,55,56,59^ The remaining 9 reported on different challenges with POC testing implementation (Table 3).^4,27,46,48,53,54,57,58^

**TABLE 3a:** Study findings.

Item	Facilitators	Reference	Barriers	Reference
Policy	None reported.	-	Absence of POC testing curriculum for professional.	45
			Absence of national policies and guidelines.	55,57
Material resources	Adequate supply of consumables.	54	Low availability.	53
	Easy to use.	58	Shortage of logistics/consumables.	56,58
	Rapid results	46,51	Lack of affordable diagnostic test kits.	57
Training needs	Refresher training.	54	Absence of training staff on POC testing.	57
	Provider expertise.	50	Lack of counselling.	49
			Absence of continuous purofessional development.	45
Commitment/ participation	Enthusiaam.	50	Lack of staff leadership involvement in POC management program.	45,51
	Political environment.	58	“I do not want to know”.	49
			Lack of public education.	57
Funding	None reported.	-	Lack of funding for training of staff.	57
			Lack of funding for procurement of logistics.	55
Human resource	None reported.	-	Inadequate human resources.	54,56
			Increase workload of professionals.	46,48,51,52,58
			Resource, and workflow disruption.	48
			High administrative burden.	59
Relationship	Coordination.	54	Poor communication.	4
			Lack of trust between staff group.	4
			Fear of disclosure regarding confidential issues.	52
			Stigmatisation.	52
Motivation	Enhanced patients’ motivation.	51	Lack of motivation.	55
Accessibility	None reported.	-	Poor access to information/low patient awareness.	48,51
Acceptability	High patients ‘ acceptability.	47,50,56	None reported.	-

POC, point-of-care.

**TABLE 3b:** Study findings.

Item	Recommendation	Reference
Policy	Strategic location and timing of testing.	52
	Technical support negotiating revisions to existing guidelines and algorithms.	55
	Effort on how the POC test might fit into existing patient workflows with minimum disruption.	47
Material resources	Consistent supply of POC test.	4
	Provision of infrastructural and good clinical management will facilitate POC implementation.	46
	Improvement in supply chain logistics management.	55
	Adequate resources.	54
	Best use of resources, which include a broader view of cost-effectiveness.	47
Training needs	Staff refresher training.	4,55
	Pain management.	49
Commitment/ participation	Continual collaboration among all POC diagnostics stakeholders in the development of an accessible curriculum to improve providers’ competence.	45
	Engagement from trusted community leaders and health providers.	52
	Community sensitization.	48,52
	Patient engagement.	4
	Political obligation.	48
	Engaging multi-level key stakeholders.	51
Funding	Adequate funding.	55

POC, point-of-care.

### Point-of-care test for use in health facilities without laboratories

**FIGURE 2:**
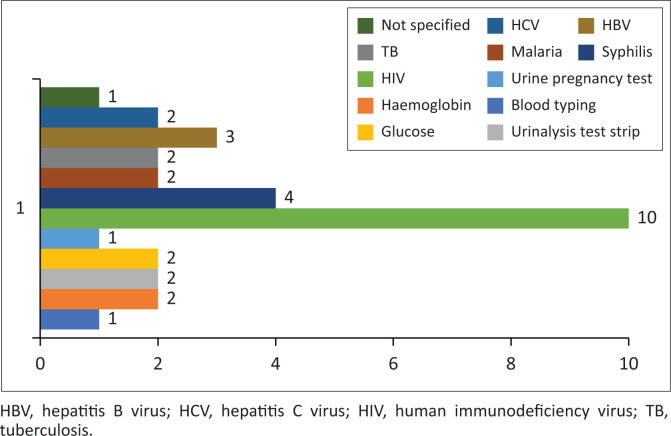
Types and number of *in vitro* diagnostics reported.

Of the 16 included studies, only two studies reported on five types of general POC tests: blood typing, haemoglobin, urinalysis, glucose and urine pregnancy test among the numerous general IVDs for use in health facilities without laboratories.^27,54^ Six types of disease-specific IVDs were documented by 10 studies,^27,46,47,50,51,52,53,56,57,59^ and one study did not specify the type of IVD^47^ See (Figure 2).

### Barriers to point-of-care test implementation

Although some barriers were country-specific, other countries in the included studies reported similar barriers. Human resource issues, such as the increased workload of healthcare professionals^46,48,51,52,58^ and inadequate human resources,^54,56^ were reported as major barriers to POC test implementation by some included studies. Again, the low availability of POC tests or inadequate resources were cited as other barriers to the implementation of POC testing services by some studies.^48,51,54,55,57^ Chamane et al. study from South Africa, Reipold et al. study on 23 LMICs, and Maddox et al. study from Malawi documented a lack of policy guidelines to regulate POC test implementation.^46,56,58^ Furthermore, insufficient funding to support staff training and procurement of logistics and supplies were unveiled by both Reipold et al. study from 23 countries in the LMICs and Maddox et al. study from Malawi. It is well noted that participation enhances commitment; a study from South Africa and Kenya reported lack of leadership and staff involvement in POC implementation as a barrier.^46,52^ Unlike the former, low patient awareness was the challenge with the POC test implementation.^49,58^ Chamane et al. also documented the absence of a POC testing curriculum as well as the lack of training and continuous professional development for healthcare workers as some of the implementation challenges. Although workflow disruption and increased administrative burden should have been seen as common barriers to POC testing implementation, these were reported by Van Hecke et al.^48^ and Bocoum et al.,^59^ respectively, from South Africa. According to a study from Ghana by Palmer et al., poor communication and lack of trust between groups were seen as barriers to POC test implementation.^4^ Lack of counseling, pain and ‘I do not want to know’ were the barriers presented by Rao et al. study from South Africa, which hinder the implementation of HIV POC testing services.^50^ Finally, Macharia et al. reported in Kenya that fear of disclosure, HIV stigma and confidentiality are the main barriers to POC implementation.^53^

### Facilitators to point-of-care test implementation

Among the included studies, Reddy et al., Bocoum et al., Rao et al. and Maddox et al. reported that the high acceptability of the POC test is a major facilitator for its implementation.^47,50,56^ According to Rao et al. and Wexler et al., POC test implementation is influenced by its rapid result which renders a short waiting time for patients.^50,52^ Moreover, according to Mohamed et al., proper coordination among stakeholders, adequate supply of consumables and frequent refresher courses for providers enable POC test implementation in the LMICs.^55^ Similar to Mohamed et al. study, Wexler et al. reported provider expertise and enthusiasm as enablers of POC test implementation.^51^ Of all the studies, peculiar reports such as easy assessment of disease progression^47^ and patient motivation^51^ were part of the facilitators of POC test implementation by Reddy et al. and Wexler respectfully. Furthermore, Bocoum et al. from Burkina Faso documented the political environment and easy use of the POC test as facilitators for its implementation.^59^

## Discussion

This study was carried out to describe existing literature on barriers and facilitators to diagnostic POC testing implementation at health facilities without laboratories in LMICs. We found 16 studies from 27 countries reporting on POC diagnostics in LMICs. These 27 countries include Brazil, Bulgaria, Burkina Faso, Burma, Cambodia, China, Egypt, Georgia, Ghana, India, Indonesia, Kenya, Macedonia, Malawi, Malaysia, Mali, Nigeria, Pakistan, Papua New Guinea, Peru, Serbia, South Africa, Turkey, Uganda, Vietnam, Zambia and Zimbabwe. The study result indicated fewer (37%) reports on the facilitators to POC diagnostics^46,47,49,50,51,54^ than barriers (63%).^4,27,45,47,48,52,53,56,57,58^ We discovered limited literature reporting on both facilitators and barriers to POC diagnostics test implementation in the LMICs, especially concerning general POC diagnostic tests for using health facilities without laboratories.

Existing literature on POC testing services implementation focused on demand-side barriers and facilitators of POC testing in advanced countries. These studies revealed patients’ acceptability and healthcare professional use of POC tests in clinical medicine in PHC facilities.^32,36^

POC testing is a vital component of the health system concerning accurate diagnosis, monitoring and screening. Though the WHO does not give specifications on the minimal performance for different POC tests, it is recommended that the safety and performance that meet quality standards should be considered by the WHO ASSURED.^25,27,33,44^ Again, the WHO recommends a country-specific design of IVDs that meet each country’s epidemiological burden.^9,10^ It is, therefore, imperative to investigate through primary research the barriers and facilitators to implementing diagnostic POC testing in resource-limited settings in LMICs. In addition, to achieve the SDG 3.8 (universal health coverage) target, it is very necessary to explore facilitators to POC diagnostic testing in the resource-limited settings in the LMICs since it has a strong bearing on the improvement and strengthening of the healthcare system in diagnosis, monitoring and treatment. Though studies were found in 27 LMICs, evidence was found from only six studies with regards to facilitators, which include coordination, adequate supply of consumables, refresher training programmes, enhanced patients’ motivation, provider enthusiasm and expertise, political environment and high acceptability. Notwithstanding, included studies revealed that low availability of POC tests concern about confidentiality, policy guidelines, inadequate funding to support staff training, poor supply chain management, poor communication, lack of staff involvement and leadership participation in POC management programmes, and absence of continuous professional development were major barriers to POC testing services implementation in the rural areas. Again, out of the 18 existing POC diagnostic tests recommended by the WHO for use in health facilities without laboratories, no studies were found on POC tests such as CD4 cell enumeration, ketones, albumin, bilirubin and white blood lactate.

### Implication for practice

The study findings imply that all rural areas in the LMICs have various challenges impeding POC testing implementation and its sustainability, which render quality of care below standard. The low availability of POC tests might have contributed to poor accessibility in the resource-limited settings in LMICs. This challenge may also result in referrals and the distraction of workflow. Thus, patients travel from their local communities to bigger facilities for some POC tests not available in their healthcare setting. It also implies more spending because of travel costs and sometimes additional costs on healthcare aside subjecting patients to more risk on the travel route. Moreover, low availability will imply that presumptive treatment, wrongful diagnosis and poor health outcomes will be high. Again, barriers such as the absence of training of staff and lack of staff involvement in POC management programmes may deny healthcare providers the professional skills in counseling and handling confidential information. Conversely, patients’ trust in health workers on confidential matters will be jeopardised. Human resource challenges might have resulted from inadequate funding to support the training of staff, which, consequently, might have contributed to a high workload of staff, poor supply chain management and an increase in administrative burden. We, therefore, recommend further studies to evaluate potential solutions to address the barriers to the POC diagnostic test implementation in resource-limited settings towards optimising the well-being of the individual and achieving SDG 3.8 (universal health coverage).

### Implication for research

The study shows limited primary research investigating the WHO EDL and POC test services in the LMICs. A sustainable POC test service will enhance accurate diagnosis and the improved well-being of individuals. Therefore, more primary research focusing on POC test implementation is recommended to explore the various facilitators of POC testing that increase service accessibility, especially in remote areas. We also recommend enhanced research to understand the challenges to POC testing implementation, particularly in the POC tests such as CD4 cell enumeration, albumin, ketones, bilirubin and white blood lactate, and thereby find potential solutions to those barriers.

### Strengths and limitations

A scoping review allows the inclusion of various study designs and enables the researcher to systematically search for and choose relevant literature to describe the evidence on a study topic. In this regard, we were able to search for relevant literature and give insight into facilitators and barriers to POC test services in the LMICs. This study also allowed us to establish literature gaps which will be useful to update forthcoming research. Earnestly, this study is the first of its kind to identify literature aiming at the WHO EDL and POC test service implementation in the resource-limited settings in the LMICs. Despite the numerous strengths, the study has many limitations. The study might have missed some relevant literature because few databases were employed for data searching. Moreover, the study was limited to PHC in the LMICs. Therefore, relevant studies might have been published in other facilities and advanced countries. Again, the study was limited to only articles published on the WHO EDL POC diagnostic test, which does not allow a review of other POC tests. Notwithstanding, the study is still important to guide future research.

## Conclusion

The study presented limited evidence of publication on the implementation of the general POC test for use in a resource-limited setting in the LMICs. It shows a research gap in general POC diagnostic tests for using health facilities without laboratories in the LMICs. Low availability of POC tests, funding and inadequate human resources remain as barriers to POC testing service implementation. It is, therefore, necessary to scale up POC testing service, particularly across rural areas of LMICs towards improving the service delivery.

## Appendix 1

**TABLE 1-A1:** Databases search results.

Date	Type of database	Keywords	Search	No. of eligible studies
03/11/2021	Google Scholar	Facilitators AND barriers AMD point-of-care diagnostics OR point-of-care services AND Implementation AND lower-and-middle income countries	2680	31
05/11/2021	EBSCOhost (Academic Search Complete)	Facilitators AND barriers AND point-of-care diagnostics OR in-vitro diagnostics AND implementation	10	4
06/11/2021	ScienceDirect	Facilitators AND barriers AND point-of-care diagnostics AND implementation AND LMICs	351	8
07/11/2021	Web of Science	Barriers OR challenge AND facilitators AND point-of-care AND implementation AND Lower-and-middle-income countries	48	10
27/02/2022	Google Scholar	Point-of-care OR point of care OR POC AND implementation AND facilitators OR enablers AND barriers OR challenges	23,300,000	7
07/03/2022	PubMed	(((((((facilitators[All Fields] OR enablers[All Fields]) AND barriers[All Fields]) OR “challenges” [All Fields]) AND (“point-of-care testing”[All Fields] OR (“point-of-care”[All Fields] AND “testing” [All Fields]) OR “point-of-care testing” [All Fields] OR (“point” [All Fields] AND’”care” [All Fields] AND “test”[All Fields]) OR “point of care test” [All Fields])) OR (“diagnostic techniques and procedures” [All Fields] OR (“diagnostic’’[All Fields] AND “techniques” [All Fields] AND “procedures” [All Fields]) OR “diagnostic techniques and procedures “[All Fields] OR (“diagnostic” [All Fields] AND “testing” [All Fields]) OR “diagnostic testing” [All Fields])) OR ((“point-of-care systems” [MeSH Terms] OR (“point-of-care”[All Fields] AND “systems”[All Fields]) OR “point-of-care systems” [All Fields] OR (“point” [All Fields] AND “care”[All Fields]) OR “point of care”[AU Fields]) AND (“technology” [MeSH Terms] OR “technology” [All Fields] OR “technologies” [All Fields]))) OR (POC[All Fields] AND (“research design” [MeSH Terms] OR (“research” [All Fields] AND “design”[All Fields]) OR “research design” [All Fields] OR “test”[All Fields]))) AND (lower-and-middle-income [All Fields] AND countries[All Fields])	897	12

**Total**	**-**	**-**	**3,209**	**72**
